# *cis*-Bis(2,2′-bipyridine-κ^2^ *N*,*N*′)bis­(dimethyl sulfoxide-κ*O*)zinc bis­(tetra­phenyl­borate) dimethyl sulfoxide monosolvate

**DOI:** 10.1107/S1600536811048082

**Published:** 2011-11-19

**Authors:** Stefania Tomyn, Elżbieta Gumienna-Kontecka, Natalia I. Usenko, Turganbay S. Iskenderov, Elena V. Prisyazhnaya

**Affiliations:** a National Taras Shevchenko University of Kyiv, Department of Chemistry, Volodymyrska Str. 64, 01601 Kyiv, Ukraine; b University of Wrocław, Faculty of Chemistry, F. Joliot-Curie Str. 14, 50-383 Wrocław, Poland; c Kyiv National University of Construction and Architecture, Department of Chemistry, Povitroflotsky Ave. 31, 03680 Kyiv, Ukraine

## Abstract

In the mononuclear title complex, [Zn(C_10_H_8_N_2_)_2_(C_2_H_6_OS)_2_](C_24_H_20_B)_2_·C_2_H_6_OS, the Zn^II^ ion is coordinated by four N atoms of two bidentate 2,2′-bipyridine mol­ecules and by the O atoms of two *cis*-disposed dimethyl sulfoxide mol­ecules in a distorted octa­hedral geometry. The S atom and the methyl groups of one of the coordinated dimethyl sulfoxide mol­ecules are disordered in a 0.509 (2):0.491 (2) ratio. The crystal packing is stabilized by C—H⋯O hydrogen bonds between the dimethyl sulfoxide solvent mol­ecules and tetra­phenyl­borate anions.

## Related literature

For uses of 2,2′-bipyridine, see: Fritsky *et al.* (1998 ▶, 2004 ▶); Kanderal *et al.* (2005 ▶); Moroz *et al.* (2010 ▶); Penkova *et al.* (2009 ▶). For related structures, see: Fritsky *et al.* (2001 ▶); Krämer & Fritsky (2000 ▶); Lalioti *et al.* (1998 ▶); Persson (1982 ▶); Petrusenko *et al.* (1997 ▶); Sachse *et al.* (2008 ▶); Wörl *et al.* (2005 ▶).
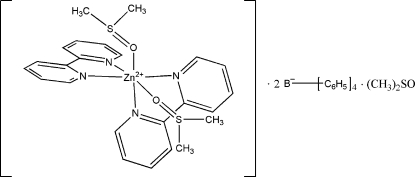


## Experimental

### 

#### Crystal data

[Zn(C_10_H_8_N_2_)_2_(C_2_H_6_OS)_2_](C_24_H_20_B)_2_·C_2_H_6_OS
*M* *_r_* = 1250.54
Monoclinic, 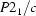

*a* = 13.8836 (5) Å
*b* = 18.0029 (7) Å
*c* = 26.1594 (10) Å
β = 90.225 (3)°
*V* = 6538.4 (4) Å^3^
*Z* = 4
Mo *K*α radiation
μ = 0.52 mm^−1^
*T* = 120 K
0.38 × 0.25 × 0.13 mm

#### Data collection

Nonius KappaCCD diffractometer
Absorption correction: multi-scan (*DENZO*/*SCALEPACK*; Otwinowski & Minor, 1997 ▶) *T*_min_ = 0.955, *T*_max_ = 0.987
44646 measured reflections
15166 independent reflections
9478 reflections with *I* > 2σ(*I*)
*R*_int_ = 0.044

#### Refinement

*R*[*F* ^2^ > 2σ(*F* ^2^)] = 0.042
*wR*(*F* ^2^) = 0.095
*S* = 0.94
15166 reflections
820 parameters
6 restraints
H-atom parameters constrained
Δρ_max_ = 0.42 e Å^−3^
Δρ_min_ = −0.48 e Å^−3^

### 

Data collection: *COLLECT* (Nonius, 2000 ▶); cell refinement: *DENZO*/*SCALEPACK* (Otwinowski & Minor, 1997 ▶); data reduction: *DENZO*/*SCALEPACK*; program(s) used to solve structure: *SIR2004* (Burla *et al.*, 2005 ▶); program(s) used to refine structure: *SHELXL97* (Sheldrick, 2008 ▶); molecular graphics: *DIAMOND* (Brandenburg, 2009 ▶); software used to prepare material for publication: *SHELXL97*.

## Supplementary Material

Crystal structure: contains datablock(s) global, I. DOI: 10.1107/S1600536811048082/zl2419sup1.cif

Structure factors: contains datablock(s) I. DOI: 10.1107/S1600536811048082/zl2419Isup2.hkl

Additional supplementary materials:  crystallographic information; 3D view; checkCIF report

## Figures and Tables

**Table 1:** Hydrogen-bond geometry (Å, °)

*D*—H⋯*A*	*D*—H	H⋯*A*	*D*⋯*A*	*D*—H⋯*A*
C23—H23⋯O3^i^	0.93	2.41	3.336 (3)	173
C59—H59⋯O3^i^	0.93	2.53	3.403 (3)	156

Symmetry code: (i) 
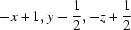
.

**Fig. 1.**
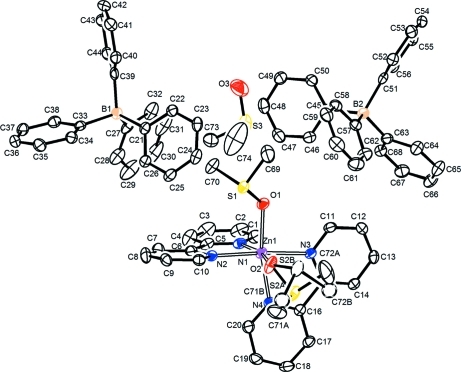
The crystal structure of the title compound. Displacement ellipsoids are drawn at the 50% probability level. H–atoms are omitted for clarity. The S atoms and methyl groups of one coordinated dimethyl sulfoxide molecule are disordered in a ratio of 0.509 (2):0.491 (2).

**Fig. 2.**
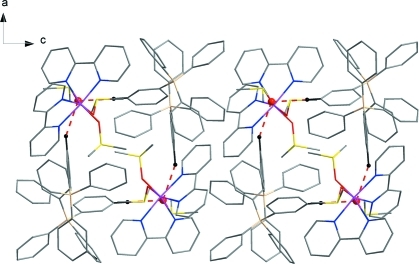
The crystal packing of the title compound viewed along [010]. H–atoms not involved in H—bonds and disordered atoms are omitted for clarity.

## supplementary crystallographic information

### Comment

2,2'-Bipyridine and 1,10-phenantroline are chelating ligands which are widely used in coordination chemistry, in particular, for the preparation of mixed ligand complexes (Fritsky *et al.*, 1998; Kanderal *et al.*, 2005). They are also often used in the synthesis of discrete polynuclear complexes in order to prevent formation of coordination polymers by blocking a certain number of vacant sites in the coordination sphere of a metal ion (Fritsky *et al.*, 2004; Moroz *et al.*, 2010; Penkova *et al.*, 2009). The title compound was obtained unintentionally as the product of an attempted synthesis of a pyrazolate complex of zinc (II) using 2,2'-bipyridine as an additional ligand and sodium tetraphenylborate for better crystallization of the resulting product.

The title compound, [Zn(C_10_H_8_N_2_)_2_(C_2_H_6_SO)_2_][(C_24_H_20_B)_2_].C_2_H_6_SO, consists of a cationic Zn^2+^ complex, two tetraphenylborate anions and a solvent dimethyl sulfoxide (DMSO) molecule. The Zn^II^ center is coordinated by four nitrogen atoms of two bidentately coordinated molecules of 2,2'-dipyridine and by the oxygen atoms of two *cis*-disposed dimethyl sulfoxide molecules. In one coordinated molecule of DMSO the S atom and the methyl groups are disordered with site occupansies of 0.5086 (17) and 0.4914 (17). The central atom adopts a highly distorted octahedral geometry with bond angles of 77.02 (6)–100.08 (6)°. The Zn—O bond lengths in the coordination sphere differ noticeably: the Zn—O1 = 2.0671 (13) Å bond is shorter and the Zn—O2 = 2.1814 (15) Å bond is much longer. Similar differences have been observed before in the related zinc complexes (Lalioti *et al.*, 1998; Persson, 1982; Petrusenko *et al.*, 1997). The Zn—N bond distances fall in the range 2.105 (2)–2.160 (2) Å. The two dipyridine molecules are nearly planar with dihedral angles of -0.9 (3)° and 5.7 (3)° between two bonded pyridine rings. The C—C and C—N bond lengths in the bipyridine molecules exhibit normal values (Fritsky *et al.*, 2001; Krämer & Fritsky, 2000; Sachse *et al.*, 2008; Wörl *et al.*, 2005). In the crystal packing the tetraphenylborate anions are linked with the solvate DMSO molecule by C—H···O hydrogen bonds and form layers coplanar the [101] plane.

### Experimental

The title compound was obtained accidentally as the product of an attempted synthesis of a zinc complex with 3,5-dimethylpyrazole, using slow evaporation of a dimethyl sulfoxide solution of zinc (II) chloride, sodium tetraphenylborate, 2,2'-bipyridine and 3,5-dimethylpyrazole at room temperature. A solution of ZnCl_2_.4H_2_O (0.208 g, 1 mmol) in DMSO (10 ml) was added to a mixture of 2,2'-bipyridine (0.312 g, 2 mmol) and 3,5-dimethylpyrazole (0.192 g, 2 mmol) in DMSO (10 ml). The mixture was stirred for 15 minutes at room temperature and filtered off. Then sodium tetraphenylborate (0.684 g, 2 mmol) in 5 ml of DMSO was added. The resulting mixture was stirred for 15 minutes, filtered off and set aside at room temperature. Colourless crystals of the title compound suitable for the X-ray analysis were obtained in three days (yield 0.837 g, 66.9%). Analysis calculated for C_74_H_74_B_2_N_4_O_3_S_3_Zn: C 71.07, H 5.96, N 4.48%, found C 70.92, H 5.84, N 4.39%.

### Refinement

The S atoms and methyl groups of one coordinated dimethylsulfoxide molecule were disordered. This was modelled with two different orientations and from refinement the site occupancies were 0.5086 (17):0.4914 (17). The highest positive peak on the residual map (σ = 0.05 e/Å^3^) is equal to 0.42 e/Å^3^ (0.18 Å from S3) and the deepest hole is -0.48 e/Å^3^ (0.74 Å from S3). All H atoms of metyl groups were positioned geometrically and refined using a riding model, with C—H = 0.96 Å and with *U*_iso_(H) = 1.5×*U*_eq_(C). All H atoms of dipyridine and phenyl rings were positioned geometrically and refined using a riding model, with C—H = 0.93 Å and with *U*_iso_(H) = 1.2×*U*_eq_(C).

### Crystal data

**Table:**

[Zn(C_10_H_8_N_2_)_2_(C_2_H_6_OS)_2_](C_24_H_20_B)_2_·C_2_H_6_OS	*F*(000) = 2632
*M**_r_* = 1250.54	*D*_x_ = 1.270 Mg m^−^^3^
Monoclinic, *P*2_1_/*c*	Mo *K*α radiation, λ = 0.71073 Å
Hall symbol: -P 2ybc	Cell parameters from 4578 reflections
*a* = 13.8836 (5) Å	θ = 3.0–27.5°
*b* = 18.0029 (7) Å	µ = 0.52 mm^−^^1^
*c* = 26.1594 (10) Å	*T* = 120 K
β = 90.225 (3)°	Block, colourless
*V* = 6538.4 (4) Å^3^	0.38 × 0.25 × 0.13 mm
*Z* = 4	

### Data collection

**Table:**

Nonius KappaCCD diffractometer	15166 independent reflections
Radiation source: fine-focus sealed tube	9478 reflections with *I* > 2σ(*I*)
horizontally mounted graphite crystal	*R*_int_ = 0.044
Detector resolution: 9 pixels mm^-1^	θ_max_ = 28.5°, θ_min_ = 3.0°
φ scans and ω scans with κ offset	*h* = −18→18
Absorption correction: multi-scan (*DENZO*/*SCALEPACK*; Otwinowski & Minor, 1997)	*k* = −23→20
*T*_min_ = 0.955, *T*_max_ = 0.987	*l* = −27→33
44646 measured reflections	

### Refinement

**Table:**

Refinement on *F*^2^	Primary atom site location: structure-invariant direct methods
Least-squares matrix: full	Secondary atom site location: difference Fourier map
*R*[*F*^2^ > 2σ(*F*^2^)] = 0.042	Hydrogen site location: inferred from neighbouring sites
*wR*(*F*^2^) = 0.095	H-atom parameters constrained
*S* = 0.94	*w* = 1/[σ^2^(*F*_o_^2^) + (0.0455*P*)^2^] where *P* = (*F*_o_^2^ + 2*F*_c_^2^)/3
15166 reflections	(Δ/σ)_max_ = 0.001
820 parameters	Δρ_max_ = 0.42 e Å^−^^3^
6 restraints	Δρ_min_ = −0.48 e Å^−^^3^

### Special details

**Table:**

Experimental. One of the coordinated DMSO molecules was found to be disordered over two positions, its sulfur and carbon atoms were refined with occupancy factors 0.502/0.498.
Geometry. All esds (except the esd in the dihedral angle between two l.s. planes) are estimated using the full covariance matrix. The cell esds are taken into account individually in the estimation of esds in distances, angles and torsion angles; correlations between esds in cell parameters are only used when they are defined by crystal symmetry. An approximate (isotropic) treatment of cell esds is used for estimating esds involving l.s. planes.
Refinement. Refinement of F^2^ against ALL reflections. The weighted R-factor wR and goodness of fit S are based on F^2^, conventional R-factors R are based on F, with F set to zero for negative F^2^. The threshold expression of F^2^ > 2sigma(F^2^) is used only for calculating R-factors(gt) etc. and is not relevant to the choice of reflections for refinement. R-factors based on F^2^ are statistically about twice as large as those based on F, and R- factors based on ALL data will be even larger.

### Fractional atomic coordinates and isotropic or equivalent isotropic displacement parameters (Å^2^)

**Table:**

	*x*	*y*	*z*	*U*_iso_*/*U*_eq_	Occ. (<1)
Zn1	0.724530 (16)	0.214565 (13)	0.146456 (9)	0.02207 (7)	
S1	0.52919 (4)	0.26321 (3)	0.20025 (2)	0.02365 (12)	
S3	0.80680 (5)	0.72158 (4)	0.11760 (3)	0.04729 (18)	
O1	0.63747 (9)	0.27658 (7)	0.19410 (5)	0.0258 (3)	
O2	0.67453 (10)	0.11039 (8)	0.17966 (6)	0.0354 (4)	
O3	0.74093 (14)	0.78001 (12)	0.14009 (7)	0.0691 (6)	
N1	0.74779 (12)	0.31086 (9)	0.09891 (7)	0.0237 (4)	
N2	0.61621 (11)	0.20371 (9)	0.09039 (6)	0.0203 (4)	
N3	0.84193 (11)	0.22497 (9)	0.19728 (6)	0.0203 (4)	
N4	0.83624 (12)	0.15402 (9)	0.10881 (6)	0.0235 (4)	
C1	0.81369 (15)	0.36383 (12)	0.10584 (9)	0.0299 (5)	
H1	0.8556	0.3595	0.1335	0.036*	
C2	0.82311 (16)	0.42462 (12)	0.07435 (10)	0.0383 (6)	
H2	0.8699	0.4605	0.0805	0.046*	
C3	0.76070 (17)	0.43066 (13)	0.03322 (11)	0.0461 (7)	
H3	0.7651	0.4709	0.0110	0.055*	
C4	0.69177 (16)	0.37650 (13)	0.02536 (9)	0.0382 (6)	
H4	0.6494	0.3799	−0.0021	0.046*	
C5	0.68637 (15)	0.31702 (12)	0.05881 (8)	0.0272 (5)	
C6	0.61165 (14)	0.25696 (11)	0.05425 (8)	0.0218 (5)	
C7	0.54197 (16)	0.25689 (13)	0.01688 (8)	0.0326 (5)	
H7	0.5414	0.2931	−0.0085	0.039*	
C8	0.47225 (16)	0.20144 (13)	0.01774 (8)	0.0323 (5)	
H8	0.4238	0.2008	−0.0069	0.039*	
C9	0.47470 (14)	0.14803 (12)	0.05463 (8)	0.0251 (5)	
H9	0.4283	0.1108	0.0557	0.030*	
C10	0.54796 (14)	0.15072 (11)	0.09034 (8)	0.0230 (5)	
H10	0.5503	0.1143	0.1155	0.028*	
C11	0.83799 (15)	0.26015 (11)	0.24241 (8)	0.0243 (5)	
H11	0.7794	0.2797	0.2530	0.029*	
C12	0.91734 (15)	0.26855 (11)	0.27372 (8)	0.0252 (5)	
H12	0.9121	0.2918	0.3053	0.030*	
C13	1.00477 (15)	0.24159 (11)	0.25703 (8)	0.0247 (5)	
H13	1.0598	0.2478	0.2770	0.030*	
C14	1.01016 (14)	0.20537 (11)	0.21058 (8)	0.0220 (5)	
H14	1.0687	0.1871	0.1989	0.026*	
C15	0.92685 (14)	0.19666 (10)	0.18153 (7)	0.0184 (4)	
C16	0.92294 (14)	0.15331 (10)	0.13289 (8)	0.0202 (4)	
C17	0.99905 (14)	0.11158 (11)	0.11420 (8)	0.0246 (5)	
H17	1.0584	0.1121	0.1308	0.029*	
C18	0.98597 (15)	0.06919 (12)	0.07069 (9)	0.0304 (5)	
H18	1.0360	0.0402	0.0581	0.036*	
C19	0.89800 (15)	0.07036 (12)	0.04606 (9)	0.0312 (5)	
H19	0.8877	0.0425	0.0166	0.037*	
C20	0.82551 (15)	0.11386 (12)	0.06609 (9)	0.0306 (5)	
H20	0.7665	0.1153	0.0491	0.037*	
C21	0.19753 (13)	0.32486 (11)	0.14914 (8)	0.0203 (5)	
C22	0.20579 (14)	0.33028 (11)	0.20236 (8)	0.0223 (5)	
H22	0.1846	0.3735	0.2182	0.027*	
C23	0.24422 (14)	0.27406 (12)	0.23266 (8)	0.0251 (5)	
H23	0.2484	0.2803	0.2679	0.030*	
C24	0.27621 (14)	0.20897 (12)	0.21053 (8)	0.0279 (5)	
H24	0.3007	0.1708	0.2307	0.033*	
C25	0.27139 (15)	0.20142 (12)	0.15797 (9)	0.0283 (5)	
H25	0.2938	0.1583	0.1425	0.034*	
C26	0.23315 (14)	0.25828 (11)	0.12828 (8)	0.0231 (5)	
H26	0.2310	0.2521	0.0930	0.028*	
C27	0.26357 (15)	0.42973 (11)	0.09225 (9)	0.0278 (5)	
C28	0.30957 (16)	0.40473 (13)	0.04815 (9)	0.0389 (6)	
H28	0.2772	0.3716	0.0269	0.047*	
C29	0.40246 (19)	0.42752 (17)	0.03459 (12)	0.0568 (8)	
H29	0.4305	0.4093	0.0049	0.068*	
C30	0.45232 (19)	0.47648 (18)	0.06485 (15)	0.0662 (10)	
H30	0.5142	0.4914	0.0560	0.079*	
C31	0.40942 (19)	0.50331 (14)	0.10863 (13)	0.0565 (9)	
H31	0.4421	0.5369	0.1294	0.068*	
C32	0.31695 (16)	0.47995 (12)	0.12172 (10)	0.0385 (6)	
H32	0.2895	0.4987	0.1514	0.046*	
C33	0.09388 (14)	0.36593 (11)	0.06429 (7)	0.0196 (4)	
C34	0.04322 (14)	0.29878 (11)	0.06323 (8)	0.0236 (5)	
H34	0.0522	0.2653	0.0899	0.028*	
C35	−0.02016 (15)	0.27994 (12)	0.02387 (8)	0.0290 (5)	
H35	−0.0528	0.2348	0.0248	0.035*	
C36	−0.03473 (16)	0.32817 (12)	−0.01664 (8)	0.0307 (5)	
H36	−0.0764	0.3157	−0.0432	0.037*	
C37	0.01389 (15)	0.39551 (12)	−0.01688 (8)	0.0287 (5)	
H37	0.0049	0.4286	−0.0438	0.034*	
C38	0.07559 (15)	0.41336 (11)	0.02278 (8)	0.0246 (5)	
H38	0.1066	0.4591	0.0219	0.030*	
C39	0.09334 (14)	0.45402 (11)	0.14258 (8)	0.0231 (5)	
C40	0.02083 (15)	0.42923 (12)	0.17619 (8)	0.0260 (5)	
H40	0.0167	0.3788	0.1835	0.031*	
C41	−0.04465 (16)	0.47739 (13)	0.19885 (8)	0.0331 (6)	
H41	−0.0918	0.4588	0.2206	0.040*	
C42	−0.03988 (18)	0.55261 (14)	0.18919 (9)	0.0387 (6)	
H42	−0.0831	0.5850	0.2046	0.046*	
C43	0.02955 (17)	0.57935 (12)	0.15653 (9)	0.0348 (6)	
H43	0.0328	0.6299	0.1495	0.042*	
C44	0.09491 (16)	0.53073 (12)	0.13393 (8)	0.0278 (5)	
H44	0.1415	0.5501	0.1122	0.033*	
C45	0.67243 (14)	0.46433 (11)	0.32789 (7)	0.0204 (4)	
C46	0.69461 (15)	0.43942 (12)	0.27845 (8)	0.0274 (5)	
H46	0.7377	0.4002	0.2750	0.033*	
C47	0.65573 (16)	0.47026 (13)	0.23464 (8)	0.0318 (5)	
H47	0.6740	0.4525	0.2027	0.038*	
C48	0.58957 (16)	0.52754 (12)	0.23819 (8)	0.0311 (5)	
H48	0.5629	0.5485	0.2089	0.037*	
C49	0.56395 (16)	0.55301 (12)	0.28611 (8)	0.0302 (5)	
H49	0.5190	0.5910	0.2892	0.036*	
C50	0.60518 (14)	0.52191 (11)	0.32977 (8)	0.0245 (5)	
H50	0.5871	0.5403	0.3615	0.029*	
C51	0.70368 (14)	0.47599 (11)	0.43037 (8)	0.0206 (4)	
C52	0.64824 (14)	0.46009 (11)	0.47371 (8)	0.0228 (5)	
H52	0.6105	0.4174	0.4738	0.027*	
C53	0.64757 (15)	0.50574 (11)	0.51668 (8)	0.0260 (5)	
H53	0.6087	0.4936	0.5443	0.031*	
C54	0.70394 (15)	0.56890 (11)	0.51879 (8)	0.0249 (5)	
H54	0.7051	0.5983	0.5480	0.030*	
C55	0.75887 (14)	0.58759 (12)	0.47635 (8)	0.0241 (5)	
H55	0.7963	0.6304	0.4766	0.029*	
C56	0.75753 (14)	0.54186 (11)	0.43360 (8)	0.0221 (5)	
H56	0.7944	0.5556	0.4055	0.027*	
C57	0.65599 (14)	0.34436 (11)	0.38358 (7)	0.0227 (5)	
C58	0.55499 (15)	0.34609 (12)	0.38794 (7)	0.0242 (5)	
H58	0.5247	0.3920	0.3905	0.029*	
C59	0.49837 (16)	0.28232 (12)	0.38860 (8)	0.0285 (5)	
H59	0.4318	0.2862	0.3914	0.034*	
C60	0.54128 (17)	0.21295 (13)	0.38513 (8)	0.0342 (6)	
H60	0.5041	0.1700	0.3861	0.041*	
C61	0.64034 (18)	0.20865 (13)	0.38029 (9)	0.0371 (6)	
H61	0.6701	0.1625	0.3777	0.045*	
C62	0.69562 (16)	0.27315 (12)	0.37923 (8)	0.0313 (5)	
H62	0.7620	0.2687	0.3755	0.038*	
C63	0.83386 (14)	0.41010 (11)	0.37502 (8)	0.0224 (5)	
C64	0.88322 (15)	0.36738 (12)	0.41138 (8)	0.0289 (5)	
H64	0.8479	0.3448	0.4372	0.035*	
C65	0.98235 (16)	0.35709 (13)	0.41071 (9)	0.0353 (6)	
H65	1.0117	0.3280	0.4357	0.042*	
C66	1.03740 (16)	0.38984 (13)	0.37327 (9)	0.0353 (6)	
H66	1.1037	0.3823	0.3723	0.042*	
C67	0.99264 (15)	0.43398 (12)	0.33710 (9)	0.0313 (5)	
H67	1.0290	0.4572	0.3120	0.038*	
C68	0.89287 (15)	0.44376 (11)	0.33823 (8)	0.0250 (5)	
H68	0.8643	0.4739	0.3136	0.030*	
C69	0.50666 (17)	0.29225 (16)	0.26363 (9)	0.0485 (7)	
H69A	0.5391	0.2596	0.2870	0.073*	
H69B	0.4386	0.2909	0.2700	0.073*	
H69C	0.5300	0.3420	0.2683	0.073*	
C70	0.47176 (15)	0.33676 (12)	0.16722 (9)	0.0335 (6)	
H70A	0.5000	0.3832	0.1774	0.050*	
H70B	0.4044	0.3370	0.1753	0.050*	
H70C	0.4797	0.3300	0.1311	0.050*	
C73	0.79903 (18)	0.73640 (16)	0.05111 (9)	0.0499 (7)	
H73A	0.7330	0.7329	0.0404	0.075*	
H73B	0.8364	0.6995	0.0337	0.075*	
H73C	0.8235	0.7849	0.0431	0.075*	
C74	0.7449 (2)	0.6357 (2)	0.11890 (16)	0.1125 (16)	
H74A	0.7351	0.6208	0.1537	0.169*	
H74B	0.7823	0.5987	0.1015	0.169*	
H74C	0.6837	0.6411	0.1021	0.169*	
B1	0.16154 (17)	0.39404 (12)	0.11232 (9)	0.0217 (5)	
B2	0.71656 (17)	0.42268 (13)	0.37894 (9)	0.0219 (5)	
S2A	0.74891 (8)	0.04806 (6)	0.18867 (4)	0.0311 (4)	0.5086 (17)
C71A	0.6866 (8)	−0.0346 (6)	0.1753 (4)	0.052 (3)	0.5086 (17)
H71D	0.6813	−0.0409	0.1390	0.079*	0.5086 (17)
H71E	0.6234	−0.0322	0.1899	0.079*	0.5086 (17)
H71F	0.7211	−0.0759	0.1897	0.079*	0.5086 (17)
C72A	0.7508 (7)	0.0468 (4)	0.2561 (2)	0.084 (3)	0.5086 (17)
H72D	0.7927	0.0079	0.2677	0.127*	0.5086 (17)
H72E	0.6868	0.0382	0.2686	0.127*	0.5086 (17)
H72F	0.7738	0.0937	0.2686	0.127*	0.5086 (17)
S2B	0.68459 (8)	0.05333 (7)	0.22553 (5)	0.0311 (4)	0.4914 (17)
C71B	0.6625 (7)	−0.0331 (5)	0.1969 (4)	0.070 (4)	0.4914 (17)
H71A	0.5976	−0.0345	0.1842	0.104*	0.4914 (17)
H71B	0.6715	−0.0718	0.2217	0.104*	0.4914 (17)
H71C	0.7065	−0.0404	0.1691	0.104*	0.4914 (17)
C72B	0.8110 (3)	0.0438 (3)	0.2349 (2)	0.0315 (13)	0.4914 (17)
H72A	0.8356	0.0873	0.2517	0.047*	0.4914 (17)
H72B	0.8420	0.0382	0.2024	0.047*	0.4914 (17)
H72C	0.8236	0.0009	0.2556	0.047*	0.4914 (17)

### Atomic displacement parameters (Å^2^)

**Table:**

	*U*^11^	*U*^22^	*U*^33^	*U*^12^	*U*^13^	*U*^23^
Zn1	0.01550 (12)	0.01983 (13)	0.03086 (15)	0.00145 (10)	−0.00370 (10)	−0.00303 (11)
S1	0.0174 (3)	0.0262 (3)	0.0274 (3)	0.0014 (2)	−0.0017 (2)	0.0019 (2)
S3	0.0360 (4)	0.0647 (5)	0.0411 (4)	−0.0026 (3)	−0.0098 (3)	0.0117 (3)
O1	0.0156 (7)	0.0286 (8)	0.0333 (9)	0.0012 (6)	−0.0006 (6)	−0.0052 (7)
O2	0.0274 (9)	0.0264 (9)	0.0523 (11)	−0.0006 (7)	−0.0144 (8)	0.0136 (7)
O3	0.0524 (13)	0.1097 (18)	0.0451 (12)	0.0091 (12)	0.0062 (10)	−0.0272 (11)
N1	0.0173 (9)	0.0204 (9)	0.0333 (11)	−0.0003 (7)	0.0032 (8)	−0.0036 (8)
N2	0.0161 (9)	0.0207 (9)	0.0242 (10)	−0.0004 (7)	0.0003 (7)	−0.0026 (7)
N3	0.0162 (9)	0.0177 (9)	0.0270 (10)	0.0006 (7)	−0.0003 (7)	−0.0023 (7)
N4	0.0201 (9)	0.0197 (9)	0.0308 (11)	0.0008 (7)	−0.0045 (8)	−0.0069 (8)
C1	0.0244 (12)	0.0228 (12)	0.0424 (14)	−0.0043 (10)	−0.0012 (10)	−0.0032 (10)
C2	0.0254 (13)	0.0244 (13)	0.0653 (18)	−0.0047 (10)	0.0067 (12)	−0.0011 (12)
C3	0.0354 (15)	0.0313 (15)	0.072 (2)	−0.0041 (11)	0.0086 (14)	0.0194 (13)
C4	0.0293 (13)	0.0395 (15)	0.0459 (15)	−0.0020 (11)	0.0003 (11)	0.0147 (12)
C5	0.0226 (12)	0.0241 (12)	0.0349 (14)	0.0038 (9)	0.0046 (10)	0.0033 (10)
C6	0.0220 (11)	0.0216 (11)	0.0217 (11)	0.0034 (9)	0.0028 (9)	0.0000 (9)
C7	0.0330 (13)	0.0361 (14)	0.0288 (13)	−0.0026 (11)	−0.0018 (10)	0.0092 (11)
C8	0.0261 (12)	0.0457 (15)	0.0249 (13)	−0.0043 (11)	−0.0075 (10)	0.0008 (11)
C9	0.0201 (11)	0.0322 (13)	0.0230 (12)	−0.0054 (9)	0.0002 (9)	−0.0048 (10)
C10	0.0229 (11)	0.0231 (11)	0.0228 (12)	0.0000 (9)	0.0029 (9)	−0.0002 (9)
C11	0.0204 (11)	0.0216 (11)	0.0310 (13)	0.0037 (9)	0.0013 (9)	−0.0044 (9)
C12	0.0270 (12)	0.0281 (12)	0.0206 (11)	0.0007 (9)	−0.0004 (9)	−0.0024 (9)
C13	0.0185 (11)	0.0307 (12)	0.0249 (12)	−0.0034 (9)	−0.0053 (9)	0.0006 (10)
C14	0.0174 (11)	0.0220 (11)	0.0266 (12)	0.0010 (9)	0.0012 (9)	0.0013 (9)
C15	0.0181 (11)	0.0138 (10)	0.0233 (11)	−0.0011 (8)	0.0001 (8)	0.0036 (8)
C16	0.0196 (11)	0.0133 (10)	0.0276 (12)	−0.0024 (8)	−0.0018 (9)	0.0010 (9)
C17	0.0171 (11)	0.0230 (12)	0.0336 (13)	0.0010 (9)	−0.0015 (9)	−0.0025 (10)
C18	0.0227 (12)	0.0256 (12)	0.0429 (15)	0.0022 (9)	0.0045 (10)	−0.0106 (11)
C19	0.0301 (13)	0.0270 (13)	0.0363 (14)	−0.0006 (10)	−0.0033 (10)	−0.0145 (10)
C20	0.0220 (12)	0.0315 (13)	0.0384 (14)	0.0006 (10)	−0.0082 (10)	−0.0115 (11)
C21	0.0142 (10)	0.0204 (11)	0.0264 (12)	−0.0039 (8)	−0.0023 (9)	0.0016 (9)
C22	0.0182 (11)	0.0217 (11)	0.0270 (12)	−0.0027 (9)	−0.0014 (9)	−0.0016 (9)
C23	0.0185 (11)	0.0325 (13)	0.0243 (12)	−0.0065 (9)	−0.0033 (9)	0.0058 (10)
C24	0.0217 (11)	0.0273 (12)	0.0346 (13)	−0.0018 (10)	−0.0062 (10)	0.0102 (10)
C25	0.0224 (12)	0.0236 (12)	0.0389 (14)	0.0032 (9)	−0.0042 (10)	0.0009 (10)
C26	0.0216 (11)	0.0221 (11)	0.0256 (12)	−0.0001 (9)	−0.0019 (9)	0.0011 (9)
C27	0.0217 (12)	0.0197 (12)	0.0419 (14)	0.0028 (9)	−0.0025 (10)	0.0113 (10)
C28	0.0286 (13)	0.0395 (15)	0.0486 (16)	0.0042 (11)	0.0019 (12)	0.0147 (12)
C29	0.0318 (15)	0.065 (2)	0.073 (2)	0.0095 (14)	0.0176 (15)	0.0388 (17)
C30	0.0209 (15)	0.059 (2)	0.119 (3)	−0.0032 (14)	0.0008 (17)	0.059 (2)
C31	0.0296 (15)	0.0306 (15)	0.109 (3)	−0.0083 (12)	−0.0241 (16)	0.0281 (16)
C32	0.0290 (13)	0.0260 (13)	0.0603 (17)	−0.0049 (11)	−0.0121 (12)	0.0113 (12)
C33	0.0190 (11)	0.0200 (11)	0.0198 (11)	0.0037 (8)	0.0031 (8)	0.0002 (9)
C34	0.0261 (12)	0.0227 (12)	0.0222 (11)	0.0007 (9)	0.0005 (9)	0.0014 (9)
C35	0.0297 (12)	0.0258 (12)	0.0315 (13)	−0.0035 (10)	−0.0035 (10)	−0.0058 (10)
C36	0.0307 (13)	0.0364 (14)	0.0249 (13)	0.0073 (11)	−0.0081 (10)	−0.0085 (10)
C37	0.0342 (13)	0.0297 (13)	0.0221 (12)	0.0101 (10)	−0.0015 (10)	0.0058 (10)
C38	0.0259 (12)	0.0202 (11)	0.0278 (12)	0.0015 (9)	0.0021 (10)	0.0026 (9)
C39	0.0233 (11)	0.0228 (12)	0.0232 (12)	−0.0010 (9)	−0.0122 (9)	−0.0034 (9)
C40	0.0250 (12)	0.0263 (12)	0.0268 (12)	0.0015 (9)	−0.0057 (10)	−0.0024 (10)
C41	0.0318 (13)	0.0422 (15)	0.0254 (13)	0.0037 (11)	−0.0017 (10)	−0.0067 (11)
C42	0.0475 (16)	0.0400 (15)	0.0287 (14)	0.0195 (12)	−0.0079 (12)	−0.0144 (11)
C43	0.0493 (16)	0.0221 (12)	0.0327 (14)	0.0115 (11)	−0.0127 (12)	−0.0054 (10)
C44	0.0349 (13)	0.0246 (12)	0.0239 (12)	0.0023 (10)	−0.0079 (10)	−0.0017 (9)
C45	0.0185 (11)	0.0197 (11)	0.0231 (11)	−0.0049 (9)	0.0015 (9)	−0.0011 (9)
C46	0.0266 (12)	0.0259 (12)	0.0298 (13)	0.0048 (9)	−0.0006 (10)	−0.0035 (10)
C47	0.0337 (13)	0.0385 (14)	0.0234 (12)	−0.0037 (11)	−0.0006 (10)	−0.0048 (10)
C48	0.0383 (14)	0.0292 (13)	0.0256 (13)	−0.0046 (11)	−0.0083 (10)	0.0055 (10)
C49	0.0353 (13)	0.0234 (12)	0.0318 (13)	0.0060 (10)	−0.0048 (10)	0.0011 (10)
C50	0.0270 (12)	0.0252 (12)	0.0213 (12)	0.0015 (9)	0.0005 (9)	−0.0011 (9)
C51	0.0168 (10)	0.0218 (11)	0.0232 (11)	0.0048 (9)	−0.0034 (9)	0.0044 (9)
C52	0.0234 (11)	0.0199 (11)	0.0253 (12)	−0.0017 (9)	−0.0007 (9)	0.0034 (9)
C53	0.0300 (12)	0.0278 (12)	0.0201 (12)	0.0019 (10)	0.0019 (9)	0.0057 (9)
C54	0.0293 (12)	0.0252 (12)	0.0203 (12)	0.0066 (9)	−0.0064 (10)	−0.0011 (9)
C55	0.0201 (11)	0.0232 (12)	0.0289 (13)	−0.0002 (9)	−0.0060 (9)	0.0011 (9)
C56	0.0157 (11)	0.0270 (12)	0.0236 (12)	0.0022 (9)	0.0003 (9)	0.0033 (9)
C57	0.0253 (12)	0.0239 (12)	0.0188 (11)	−0.0003 (9)	0.0019 (9)	−0.0003 (9)
C58	0.0291 (12)	0.0245 (12)	0.0189 (11)	0.0000 (9)	0.0027 (9)	−0.0001 (9)
C59	0.0284 (12)	0.0354 (13)	0.0218 (12)	−0.0070 (11)	0.0031 (9)	0.0011 (10)
C60	0.0443 (15)	0.0271 (13)	0.0312 (13)	−0.0125 (11)	0.0045 (11)	0.0014 (10)
C61	0.0455 (15)	0.0236 (13)	0.0423 (15)	0.0025 (11)	0.0029 (12)	−0.0007 (11)
C62	0.0277 (12)	0.0274 (13)	0.0388 (14)	0.0001 (10)	0.0037 (10)	0.0016 (10)
C63	0.0222 (11)	0.0207 (11)	0.0244 (12)	0.0023 (9)	0.0008 (9)	−0.0024 (9)
C64	0.0244 (12)	0.0321 (13)	0.0302 (13)	0.0014 (10)	0.0017 (10)	0.0072 (10)
C65	0.0270 (13)	0.0371 (14)	0.0419 (15)	0.0072 (11)	−0.0057 (11)	0.0083 (11)
C66	0.0161 (11)	0.0403 (15)	0.0495 (16)	0.0026 (10)	0.0001 (11)	0.0019 (12)
C67	0.0250 (12)	0.0343 (13)	0.0345 (14)	−0.0009 (10)	0.0070 (10)	0.0006 (11)
C68	0.0247 (12)	0.0245 (12)	0.0258 (12)	0.0027 (9)	0.0003 (9)	−0.0007 (9)
C69	0.0241 (13)	0.091 (2)	0.0309 (14)	−0.0020 (14)	0.0028 (11)	−0.0040 (14)
C70	0.0219 (12)	0.0317 (13)	0.0469 (15)	0.0042 (10)	−0.0039 (11)	0.0097 (11)
C73	0.0400 (16)	0.075 (2)	0.0352 (15)	0.0169 (14)	0.0018 (12)	−0.0133 (14)
C74	0.061 (2)	0.088 (3)	0.188 (4)	−0.029 (2)	−0.032 (3)	0.067 (3)
B1	0.0229 (13)	0.0165 (12)	0.0256 (14)	−0.0012 (10)	−0.0040 (10)	0.0019 (10)
B2	0.0201 (12)	0.0201 (13)	0.0256 (14)	0.0016 (10)	0.0000 (10)	−0.0010 (10)
S2A	0.0255 (7)	0.0288 (7)	0.0391 (8)	0.0034 (5)	−0.0019 (5)	0.0142 (5)
C71A	0.068 (6)	0.032 (4)	0.057 (5)	0.002 (3)	0.003 (4)	−0.015 (4)
C72A	0.166 (9)	0.042 (4)	0.044 (4)	0.014 (5)	−0.050 (5)	0.008 (3)
S2B	0.0233 (7)	0.0374 (7)	0.0326 (8)	0.0003 (5)	0.0000 (5)	0.0149 (5)
C71B	0.066 (6)	0.024 (4)	0.118 (10)	−0.004 (4)	−0.057 (7)	0.015 (5)
C72B	0.022 (3)	0.039 (3)	0.034 (3)	0.002 (2)	−0.004 (2)	0.015 (3)

### Geometric parameters (Å, °)

**Table:**

Zn1—O1	2.0671 (13)	C37—H37	0.9300
Zn1—N2	2.1055 (16)	C38—H38	0.9300
Zn1—N3	2.1076 (16)	C39—C44	1.399 (3)
Zn1—N4	2.1390 (16)	C39—C40	1.412 (3)
Zn1—N1	2.1586 (17)	C39—B1	1.642 (3)
Zn1—O2	2.1814 (14)	C40—C41	1.391 (3)
S1—O1	1.5316 (14)	C40—H40	0.9300
S1—C69	1.767 (2)	C41—C42	1.379 (3)
S1—C70	1.769 (2)	C41—H41	0.9300
S3—O3	1.514 (2)	C42—C43	1.377 (3)
S3—C73	1.763 (2)	C42—H42	0.9300
S3—C74	1.769 (3)	C43—C44	1.394 (3)
O2—S2A	1.5425 (17)	C43—H43	0.9300
O2—S2B	1.5854 (18)	C44—H44	0.9300
N1—C1	1.334 (2)	C45—C50	1.396 (3)
N1—C5	1.354 (3)	C45—C46	1.404 (3)
N2—C10	1.345 (2)	C45—B2	1.647 (3)
N2—C6	1.348 (2)	C46—C47	1.381 (3)
N3—C11	1.341 (2)	C46—H46	0.9300
N3—C15	1.350 (2)	C47—C48	1.384 (3)
N4—C20	1.339 (3)	C47—H47	0.9300
N4—C16	1.356 (2)	C48—C49	1.383 (3)
C1—C2	1.376 (3)	C48—H48	0.9300
C1—H1	0.9300	C49—C50	1.393 (3)
C2—C3	1.383 (3)	C49—H49	0.9300
C2—H2	0.9300	C50—H50	0.9300
C3—C4	1.381 (3)	C51—C52	1.402 (3)
C3—H3	0.9300	C51—C56	1.404 (3)
C4—C5	1.385 (3)	C51—B2	1.663 (3)
C4—H4	0.9300	C52—C53	1.393 (3)
C5—C6	1.503 (3)	C52—H52	0.9300
C6—C7	1.372 (3)	C53—C54	1.381 (3)
C7—C8	1.391 (3)	C53—H53	0.9300
C7—H7	0.9300	C54—C55	1.391 (3)
C8—C9	1.363 (3)	C54—H54	0.9300
C8—H8	0.9300	C55—C56	1.389 (3)
C9—C10	1.379 (3)	C55—H55	0.9300
C9—H9	0.9300	C56—H56	0.9300
C10—H10	0.9300	C57—C62	1.400 (3)
C11—C12	1.378 (3)	C57—C58	1.408 (3)
C11—H11	0.9300	C57—B2	1.646 (3)
C12—C13	1.380 (3)	C58—C59	1.392 (3)
C12—H12	0.9300	C58—H58	0.9300
C13—C14	1.381 (3)	C59—C60	1.387 (3)
C13—H13	0.9300	C59—H59	0.9300
C14—C15	1.390 (3)	C60—C61	1.384 (3)
C14—H14	0.9300	C60—H60	0.9300
C15—C16	1.494 (3)	C61—C62	1.392 (3)
C16—C17	1.387 (3)	C61—H61	0.9300
C17—C18	1.382 (3)	C62—H62	0.9300
C17—H17	0.9300	C63—C64	1.400 (3)
C18—C19	1.379 (3)	C63—C68	1.404 (3)
C18—H18	0.9300	C63—B2	1.648 (3)
C19—C20	1.380 (3)	C64—C65	1.389 (3)
C19—H19	0.9300	C64—H64	0.9300
C20—H20	0.9300	C65—C66	1.377 (3)
C21—C22	1.400 (3)	C65—H65	0.9300
C21—C26	1.408 (3)	C66—C67	1.381 (3)
C21—B1	1.651 (3)	C66—H66	0.9300
C22—C23	1.391 (3)	C67—C68	1.397 (3)
C22—H22	0.9300	C67—H67	0.9300
C23—C24	1.381 (3)	C68—H68	0.9300
C23—H23	0.9300	C69—H69A	0.9600
C24—C25	1.383 (3)	C69—H69B	0.9600
C24—H24	0.9300	C69—H69C	0.9600
C25—C26	1.389 (3)	C70—H70A	0.9600
C25—H25	0.9300	C70—H70B	0.9600
C26—H26	0.9300	C70—H70C	0.9600
C27—C28	1.395 (3)	C73—H73A	0.9600
C27—C32	1.399 (3)	C73—H73B	0.9600
C27—B1	1.643 (3)	C73—H73C	0.9600
C28—C29	1.400 (3)	C74—H74A	0.9600
C28—H28	0.9300	C74—H74B	0.9600
C29—C30	1.370 (4)	C74—H74C	0.9600
C29—H29	0.9300	S2A—C71A	1.756 (9)
C30—C31	1.380 (4)	S2A—C72A	1.763 (6)
C30—H30	0.9300	C71A—H71D	0.9600
C31—C32	1.395 (3)	C71A—H71E	0.9600
C31—H31	0.9300	C71A—H71F	0.9600
C32—H32	0.9300	C72A—H72D	0.9600
C33—C34	1.399 (3)	C72A—H72E	0.9600
C33—C38	1.404 (3)	C72A—H72F	0.9600
C33—B1	1.645 (3)	S2B—C71B	1.754 (8)
C34—C35	1.394 (3)	S2B—C72B	1.779 (5)
C34—H34	0.9300	C71B—H71A	0.9600
C35—C36	1.384 (3)	C71B—H71B	0.9600
C35—H35	0.9300	C71B—H71C	0.9600
C36—C37	1.388 (3)	C72B—H72A	0.9600
C36—H36	0.9300	C72B—H72B	0.9600
C37—C38	1.381 (3)	C72B—H72C	0.9600
			
O1—Zn1—N2	93.00 (6)	C37—C38—C33	123.3 (2)
O1—Zn1—N3	91.39 (6)	C37—C38—H38	118.3
N2—Zn1—N3	174.92 (6)	C33—C38—H38	118.3
O1—Zn1—N4	168.40 (6)	C44—C39—C40	115.10 (19)
N2—Zn1—N4	98.58 (6)	C44—C39—B1	124.17 (19)
N3—Zn1—N4	77.02 (6)	C40—C39—B1	120.43 (18)
O1—Zn1—N1	90.16 (6)	C41—C40—C39	122.5 (2)
N2—Zn1—N1	77.36 (6)	C41—C40—H40	118.7
N3—Zn1—N1	100.08 (6)	C39—C40—H40	118.7
N4—Zn1—N1	91.92 (6)	C42—C41—C40	120.1 (2)
O1—Zn1—O2	92.09 (6)	C42—C41—H41	119.9
N2—Zn1—O2	88.28 (6)	C40—C41—H41	119.9
N3—Zn1—O2	94.12 (6)	C43—C42—C41	119.4 (2)
N4—Zn1—O2	88.72 (6)	C43—C42—H42	120.3
N1—Zn1—O2	165.57 (6)	C41—C42—H42	120.3
O1—S1—C69	103.26 (10)	C42—C43—C44	120.1 (2)
O1—S1—C70	105.77 (9)	C42—C43—H43	119.9
C69—S1—C70	98.96 (12)	C44—C43—H43	119.9
O3—S3—C73	104.10 (12)	C43—C44—C39	122.7 (2)
O3—S3—C74	107.78 (16)	C43—C44—H44	118.6
C73—S3—C74	97.08 (17)	C39—C44—H44	118.6
S1—O1—Zn1	123.71 (8)	C50—C45—C46	114.75 (18)
S2A—O2—S2B	49.91 (7)	C50—C45—B2	123.78 (18)
S2A—O2—Zn1	118.20 (9)	C46—C45—B2	121.27 (18)
S2B—O2—Zn1	146.23 (10)	C47—C46—C45	123.3 (2)
C1—N1—C5	118.45 (18)	C47—C46—H46	118.3
C1—N1—Zn1	126.88 (15)	C45—C46—H46	118.3
C5—N1—Zn1	114.65 (13)	C46—C47—C48	120.1 (2)
C10—N2—C6	118.21 (17)	C46—C47—H47	120.0
C10—N2—Zn1	124.60 (14)	C48—C47—H47	120.0
C6—N2—Zn1	117.01 (13)	C49—C48—C47	118.8 (2)
C11—N3—C15	119.04 (17)	C49—C48—H48	120.6
C11—N3—Zn1	124.28 (13)	C47—C48—H48	120.6
C15—N3—Zn1	116.62 (13)	C48—C49—C50	120.2 (2)
C20—N4—C16	118.57 (17)	C48—C49—H49	119.9
C20—N4—Zn1	125.51 (14)	C50—C49—H49	119.9
C16—N4—Zn1	115.77 (13)	C49—C50—C45	122.8 (2)
N1—C1—C2	123.6 (2)	C49—C50—H50	118.6
N1—C1—H1	118.2	C45—C50—H50	118.6
C2—C1—H1	118.2	C52—C51—C56	114.67 (18)
C1—C2—C3	117.9 (2)	C52—C51—B2	126.69 (18)
C1—C2—H2	121.1	C56—C51—B2	118.50 (17)
C3—C2—H2	121.1	C53—C52—C51	122.52 (19)
C4—C3—C2	119.5 (2)	C53—C52—H52	118.7
C4—C3—H3	120.3	C51—C52—H52	118.7
C2—C3—H3	120.3	C54—C53—C52	120.8 (2)
C3—C4—C5	119.4 (2)	C54—C53—H53	119.6
C3—C4—H4	120.3	C52—C53—H53	119.6
C5—C4—H4	120.3	C53—C54—C55	118.65 (19)
N1—C5—C4	121.2 (2)	C53—C54—H54	120.7
N1—C5—C6	115.79 (18)	C55—C54—H54	120.7
C4—C5—C6	123.0 (2)	C56—C55—C54	119.6 (2)
N2—C6—C7	122.01 (19)	C56—C55—H55	120.2
N2—C6—C5	115.17 (18)	C54—C55—H55	120.2
C7—C6—C5	122.79 (19)	C55—C56—C51	123.68 (19)
C6—C7—C8	118.5 (2)	C55—C56—H56	118.2
C6—C7—H7	120.7	C51—C56—H56	118.2
C8—C7—H7	120.7	C62—C57—C58	114.76 (19)
C9—C8—C7	120.2 (2)	C62—C57—B2	125.26 (18)
C9—C8—H8	119.9	C58—C57—B2	119.75 (18)
C7—C8—H8	119.9	C59—C58—C57	123.1 (2)
C8—C9—C10	118.1 (2)	C59—C58—H58	118.5
C8—C9—H9	120.9	C57—C58—H58	118.5
C10—C9—H9	120.9	C60—C59—C58	120.0 (2)
N2—C10—C9	122.92 (19)	C60—C59—H59	120.0
N2—C10—H10	118.5	C58—C59—H59	120.0
C9—C10—H10	118.5	C61—C60—C59	118.9 (2)
N3—C11—C12	122.67 (19)	C61—C60—H60	120.5
N3—C11—H11	118.7	C59—C60—H60	120.5
C12—C11—H11	118.7	C60—C61—C62	120.2 (2)
C11—C12—C13	118.38 (19)	C60—C61—H61	119.9
C11—C12—H12	120.8	C62—C61—H61	119.9
C13—C12—H12	120.8	C61—C62—C57	123.1 (2)
C12—C13—C14	119.68 (19)	C61—C62—H62	118.5
C12—C13—H13	120.2	C57—C62—H62	118.5
C14—C13—H13	120.2	C64—C63—C68	114.67 (18)
C13—C14—C15	119.09 (18)	C64—C63—B2	120.96 (18)
C13—C14—H14	120.5	C68—C63—B2	124.25 (18)
C15—C14—H14	120.5	C65—C64—C63	123.2 (2)
N3—C15—C14	121.08 (18)	C65—C64—H64	118.4
N3—C15—C16	115.36 (17)	C63—C64—H64	118.4
C14—C15—C16	123.50 (17)	C66—C65—C64	120.3 (2)
N4—C16—C17	121.15 (18)	C66—C65—H65	119.8
N4—C16—C15	114.83 (17)	C64—C65—H65	119.8
C17—C16—C15	123.92 (18)	C65—C66—C67	118.9 (2)
C18—C17—C16	119.45 (19)	C65—C66—H66	120.5
C18—C17—H17	120.3	C67—C66—H66	120.5
C16—C17—H17	120.3	C66—C67—C68	120.1 (2)
C19—C18—C17	119.3 (2)	C66—C67—H67	119.9
C19—C18—H18	120.3	C68—C67—H67	119.9
C17—C18—H18	120.3	C67—C68—C63	122.8 (2)
C18—C19—C20	118.5 (2)	C67—C68—H68	118.6
C18—C19—H19	120.7	C63—C68—H68	118.6
C20—C19—H19	120.7	S1—C69—H69A	109.5
N4—C20—C19	122.98 (19)	S1—C69—H69B	109.5
N4—C20—H20	118.5	H69A—C69—H69B	109.5
C19—C20—H20	118.5	S1—C69—H69C	109.5
C22—C21—C26	114.67 (18)	H69A—C69—H69C	109.5
C22—C21—B1	123.45 (18)	H69B—C69—H69C	109.5
C26—C21—B1	121.49 (18)	S1—C70—H70A	109.5
C23—C22—C21	123.08 (19)	S1—C70—H70B	109.5
C23—C22—H22	118.5	H70A—C70—H70B	109.5
C21—C22—H22	118.5	S1—C70—H70C	109.5
C24—C23—C22	120.1 (2)	H70A—C70—H70C	109.5
C24—C23—H23	119.9	H70B—C70—H70C	109.5
C22—C23—H23	119.9	S3—C73—H73A	109.5
C23—C24—C25	119.06 (19)	S3—C73—H73B	109.5
C23—C24—H24	120.5	H73A—C73—H73B	109.5
C25—C24—H24	120.5	S3—C73—H73C	109.5
C24—C25—C26	120.0 (2)	H73A—C73—H73C	109.5
C24—C25—H25	120.0	H73B—C73—H73C	109.5
C26—C25—H25	120.0	S3—C74—H74A	109.5
C25—C26—C21	123.0 (2)	S3—C74—H74B	109.5
C25—C26—H26	118.5	H74A—C74—H74B	109.5
C21—C26—H26	118.5	S3—C74—H74C	109.5
C28—C27—C32	114.9 (2)	H74A—C74—H74C	109.5
C28—C27—B1	122.4 (2)	H74B—C74—H74C	109.5
C32—C27—B1	122.1 (2)	C39—B1—C27	113.36 (17)
C27—C28—C29	122.7 (3)	C39—B1—C33	104.00 (16)
C27—C28—H28	118.7	C27—B1—C33	111.51 (17)
C29—C28—H28	118.7	C39—B1—C21	112.87 (17)
C30—C29—C28	120.4 (3)	C27—B1—C21	102.83 (16)
C30—C29—H29	119.8	C33—B1—C21	112.58 (16)
C28—C29—H29	119.8	C57—B2—C63	113.08 (17)
C29—C30—C31	119.1 (3)	C57—B2—C45	105.14 (16)
C29—C30—H30	120.5	C63—B2—C45	112.13 (17)
C31—C30—H30	120.5	C57—B2—C51	112.21 (17)
C30—C31—C32	119.9 (3)	C63—B2—C51	103.83 (16)
C30—C31—H31	120.0	C45—B2—C51	110.63 (16)
C32—C31—H31	120.0	O2—S2A—C71A	104.9 (4)
C31—C32—C27	123.1 (3)	O2—S2A—C72A	99.7 (3)
C31—C32—H32	118.5	C71A—S2A—C72A	101.2 (4)
C27—C32—H32	118.5	O2—S2B—C71B	103.7 (4)
C34—C33—C38	114.89 (18)	O2—S2B—C72B	104.50 (19)
C34—C33—B1	124.51 (17)	C71B—S2B—C72B	98.3 (4)
C38—C33—B1	120.30 (17)	S2B—C71B—H71A	109.5
C35—C34—C33	122.75 (19)	S2B—C71B—H71B	109.5
C35—C34—H34	118.6	H71A—C71B—H71B	109.5
C33—C34—H34	118.6	S2B—C71B—H71C	109.5
C36—C35—C34	120.2 (2)	H71A—C71B—H71C	109.5
C36—C35—H35	119.9	H71B—C71B—H71C	109.5
C34—C35—H35	119.9	S2B—C72B—H72A	109.5
C35—C36—C37	118.8 (2)	S2B—C72B—H72B	109.5
C35—C36—H36	120.6	H72A—C72B—H72B	109.5
C37—C36—H36	120.6	S2B—C72B—H72C	109.5
C38—C37—C36	120.0 (2)	H72A—C72B—H72C	109.5
C38—C37—H37	120.0	H72B—C72B—H72C	109.5
C36—C37—H37	120.0		
			
C69—S1—O1—Zn1	150.41 (12)	C32—C27—C28—C29	0.6 (3)
C70—S1—O1—Zn1	−106.12 (12)	B1—C27—C28—C29	−171.0 (2)
N2—Zn1—O1—S1	36.34 (10)	C27—C28—C29—C30	−0.2 (4)
N3—Zn1—O1—S1	−146.23 (10)	C28—C29—C30—C31	−0.4 (4)
N4—Zn1—O1—S1	−145.9 (3)	C29—C30—C31—C32	0.6 (4)
N1—Zn1—O1—S1	113.69 (10)	C30—C31—C32—C27	−0.2 (4)
O2—Zn1—O1—S1	−52.05 (10)	C28—C27—C32—C31	−0.4 (3)
O1—Zn1—O2—S2A	−140.15 (11)	B1—C27—C32—C31	171.2 (2)
N2—Zn1—O2—S2A	126.91 (11)	C38—C33—C34—C35	0.4 (3)
N3—Zn1—O2—S2A	−48.61 (11)	B1—C33—C34—C35	174.04 (19)
N4—Zn1—O2—S2A	28.28 (11)	C33—C34—C35—C36	0.5 (3)
N1—Zn1—O2—S2A	121.0 (2)	C34—C35—C36—C37	−0.8 (3)
O1—Zn1—O2—S2B	−81.01 (19)	C35—C36—C37—C38	0.1 (3)
N2—Zn1—O2—S2B	−173.95 (19)	C36—C37—C38—C33	0.9 (3)
N3—Zn1—O2—S2B	10.54 (19)	C34—C33—C38—C37	−1.1 (3)
N4—Zn1—O2—S2B	87.42 (19)	B1—C33—C38—C37	−175.04 (19)
N1—Zn1—O2—S2B	−179.84 (19)	C44—C39—C40—C41	0.4 (3)
O1—Zn1—N1—C1	85.16 (17)	B1—C39—C40—C41	−173.66 (18)
N2—Zn1—N1—C1	178.20 (18)	C39—C40—C41—C42	−0.6 (3)
N3—Zn1—N1—C1	−6.28 (18)	C40—C41—C42—C43	0.7 (3)
N4—Zn1—N1—C1	−83.43 (17)	C41—C42—C43—C44	−0.7 (3)
O2—Zn1—N1—C1	−175.8 (2)	C42—C43—C44—C39	0.5 (3)
O1—Zn1—N1—C5	−93.39 (14)	C40—C39—C44—C43	−0.3 (3)
N2—Zn1—N1—C5	−0.34 (14)	B1—C39—C44—C43	173.45 (19)
N3—Zn1—N1—C5	175.18 (14)	C50—C45—C46—C47	1.8 (3)
N4—Zn1—N1—C5	98.02 (14)	B2—C45—C46—C47	176.8 (2)
O2—Zn1—N1—C5	5.7 (3)	C45—C46—C47—C48	−1.6 (3)
O1—Zn1—N2—C10	−85.83 (15)	C46—C47—C48—C49	0.2 (3)
N4—Zn1—N2—C10	94.62 (16)	C47—C48—C49—C50	0.8 (3)
N1—Zn1—N2—C10	−175.33 (16)	C48—C49—C50—C45	−0.5 (3)
O2—Zn1—N2—C10	6.17 (15)	C46—C45—C50—C49	−0.7 (3)
O1—Zn1—N2—C6	89.30 (14)	B2—C45—C50—C49	−175.57 (19)
N4—Zn1—N2—C6	−90.25 (14)	C56—C51—C52—C53	−0.6 (3)
N1—Zn1—N2—C6	−0.20 (14)	B2—C51—C52—C53	175.05 (19)
O2—Zn1—N2—C6	−178.69 (14)	C51—C52—C53—C54	−1.3 (3)
O1—Zn1—N3—C11	2.14 (16)	C52—C53—C54—C55	2.3 (3)
N4—Zn1—N3—C11	−177.79 (17)	C53—C54—C55—C56	−1.4 (3)
N1—Zn1—N3—C11	92.56 (16)	C54—C55—C56—C51	−0.5 (3)
O2—Zn1—N3—C11	−90.06 (16)	C52—C51—C56—C55	1.5 (3)
O1—Zn1—N3—C15	−174.96 (14)	B2—C51—C56—C55	−174.51 (18)
N4—Zn1—N3—C15	5.11 (13)	C62—C57—C58—C59	0.8 (3)
N1—Zn1—N3—C15	−84.54 (14)	B2—C57—C58—C59	175.60 (19)
O2—Zn1—N3—C15	92.84 (14)	C57—C58—C59—C60	0.3 (3)
O1—Zn1—N4—C20	173.3 (3)	C58—C59—C60—C61	−0.9 (3)
N2—Zn1—N4—C20	−8.94 (18)	C59—C60—C61—C62	0.4 (3)
N3—Zn1—N4—C20	173.65 (18)	C60—C61—C62—C57	0.9 (4)
N1—Zn1—N4—C20	−86.45 (18)	C58—C57—C62—C61	−1.4 (3)
O2—Zn1—N4—C20	79.13 (18)	B2—C57—C62—C61	−175.9 (2)
O1—Zn1—N4—C16	−2.2 (4)	C68—C63—C64—C65	−1.6 (3)
N2—Zn1—N4—C16	175.56 (14)	B2—C63—C64—C65	−177.9 (2)
N3—Zn1—N4—C16	−1.85 (13)	C63—C64—C65—C66	0.3 (4)
N1—Zn1—N4—C16	98.05 (14)	C64—C65—C66—C67	1.2 (4)
O2—Zn1—N4—C16	−96.37 (14)	C65—C66—C67—C68	−1.2 (3)
C5—N1—C1—C2	−0.2 (3)	C66—C67—C68—C63	−0.2 (3)
Zn1—N1—C1—C2	−178.68 (16)	C64—C63—C68—C67	1.6 (3)
N1—C1—C2—C3	−0.2 (3)	B2—C63—C68—C67	177.65 (19)
C1—C2—C3—C4	0.3 (4)	C44—C39—B1—C27	27.5 (3)
C2—C3—C4—C5	0.0 (4)	C40—C39—B1—C27	−159.10 (18)
C1—N1—C5—C4	0.5 (3)	C44—C39—B1—C33	−93.8 (2)
Zn1—N1—C5—C4	179.13 (17)	C40—C39—B1—C33	79.6 (2)
C1—N1—C5—C6	−177.89 (18)	C44—C39—B1—C21	143.85 (19)
Zn1—N1—C5—C6	0.8 (2)	C40—C39—B1—C21	−42.7 (2)
C3—C4—C5—N1	−0.4 (3)	C28—C27—B1—C39	−149.10 (19)
C3—C4—C5—C6	177.9 (2)	C32—C27—B1—C39	39.9 (3)
C10—N2—C6—C7	−2.3 (3)	C28—C27—B1—C33	−32.1 (3)
Zn1—N2—C6—C7	−177.75 (16)	C32—C27—B1—C33	156.83 (19)
C10—N2—C6—C5	176.11 (17)	C28—C27—B1—C21	88.7 (2)
Zn1—N2—C6—C5	0.7 (2)	C32—C27—B1—C21	−82.3 (2)
N1—C5—C6—N2	−1.0 (3)	C34—C33—B1—C39	−100.8 (2)
C4—C5—C6—N2	−179.3 (2)	C38—C33—B1—C39	72.5 (2)
N1—C5—C6—C7	177.43 (19)	C34—C33—B1—C27	136.68 (19)
C4—C5—C6—C7	−0.9 (3)	C38—C33—B1—C27	−50.0 (2)
N2—C6—C7—C8	2.3 (3)	C34—C33—B1—C21	21.7 (3)
C5—C6—C7—C8	−176.0 (2)	C38—C33—B1—C21	−164.99 (18)
C6—C7—C8—C9	−1.0 (3)	C22—C21—B1—C39	−25.4 (3)
C7—C8—C9—C10	−0.3 (3)	C26—C21—B1—C39	162.23 (17)
C6—N2—C10—C9	0.9 (3)	C22—C21—B1—C27	97.1 (2)
Zn1—N2—C10—C9	176.00 (14)	C26—C21—B1—C27	−75.3 (2)
C8—C9—C10—N2	0.4 (3)	C22—C21—B1—C33	−142.76 (18)
C15—N3—C11—C12	−0.2 (3)	C26—C21—B1—C33	44.9 (2)
Zn1—N3—C11—C12	−177.21 (15)	C62—C57—B2—C63	−6.7 (3)
N3—C11—C12—C13	2.1 (3)	C58—C57—B2—C63	179.14 (18)
C11—C12—C13—C14	−1.8 (3)	C62—C57—B2—C45	116.0 (2)
C12—C13—C14—C15	−0.2 (3)	C58—C57—B2—C45	−58.2 (2)
C11—N3—C15—C14	−1.9 (3)	C62—C57—B2—C51	−123.7 (2)
Zn1—N3—C15—C14	175.33 (14)	C58—C57—B2—C51	62.1 (2)
C11—N3—C15—C16	175.37 (17)	C64—C63—B2—C57	−53.1 (3)
Zn1—N3—C15—C16	−7.4 (2)	C68—C63—B2—C57	131.0 (2)
C13—C14—C15—N3	2.1 (3)	C64—C63—B2—C45	−171.78 (18)
C13—C14—C15—C16	−174.98 (18)	C68—C63—B2—C45	12.4 (3)
C20—N4—C16—C17	−0.5 (3)	C64—C63—B2—C51	68.8 (2)
Zn1—N4—C16—C17	175.30 (15)	C68—C63—B2—C51	−107.1 (2)
C20—N4—C16—C15	−177.14 (18)	C50—C45—B2—C57	100.5 (2)
Zn1—N4—C16—C15	−1.3 (2)	C46—C45—B2—C57	−74.0 (2)
N3—C15—C16—N4	5.7 (2)	C50—C45—B2—C63	−136.23 (19)
C14—C15—C16—N4	−177.10 (18)	C46—C45—B2—C63	49.2 (2)
N3—C15—C16—C17	−170.81 (18)	C50—C45—B2—C51	−20.8 (3)
C14—C15—C16—C17	6.4 (3)	C46—C45—B2—C51	164.63 (18)
N4—C16—C17—C18	−0.9 (3)	C52—C51—B2—C57	−0.6 (3)
C15—C16—C17—C18	175.40 (19)	C56—C51—B2—C57	174.88 (17)
C16—C17—C18—C19	1.3 (3)	C52—C51—B2—C63	−123.1 (2)
C17—C18—C19—C20	−0.3 (3)	C56—C51—B2—C63	52.4 (2)
C16—N4—C20—C19	1.6 (3)	C52—C51—B2—C45	116.4 (2)
Zn1—N4—C20—C19	−173.82 (16)	C56—C51—B2—C45	−68.0 (2)
C18—C19—C20—N4	−1.1 (3)	S2B—O2—S2A—C71A	75.0 (3)
C26—C21—C22—C23	−1.2 (3)	Zn1—O2—S2A—C71A	−143.6 (3)
B1—C21—C22—C23	−174.09 (18)	S2B—O2—S2A—C72A	−29.4 (3)
C21—C22—C23—C24	−0.2 (3)	Zn1—O2—S2A—C72A	112.0 (3)
C22—C23—C24—C25	1.4 (3)	S2A—O2—S2B—C71B	−65.2 (4)
C23—C24—C25—C26	−1.2 (3)	Zn1—O2—S2B—C71B	−146.7 (4)
C24—C25—C26—C21	−0.4 (3)	S2A—O2—S2B—C72B	37.3 (2)
C22—C21—C26—C25	1.5 (3)	Zn1—O2—S2B—C72B	−44.2 (3)
B1—C21—C26—C25	174.53 (19)		

### Hydrogen-bond geometry (Å, °)

**Table:**

*D*—H···*A*	*D*—H	H···*A*	*D*···*A*	*D*—H···*A*
C23—H23···O3^i^	0.93	2.41	3.336 (3)	173.
C59—H59···O3^i^	0.93	2.53	3.403 (3)	156.

Symmetry codes: (i) −*x*+1, *y*−1/2, −*z*+1/2.
